# Alterations of T Cell Subsets Associated with Sickle Cell Trait

**DOI:** 10.70322/bgd.2025.10001

**Published:** 2024-10-08

**Authors:** Alexander P. Reiner, Laura M. Raffield, Lynette Ekunwe, Nels C. Olson, Paul L. Auer, Margaret F. Doyle

**Affiliations:** 1 Department of Epidemiology, University of Washington, Seattle, WA 98105, USA; 2 Public Health Sciences Division, Fred Hutchinson Cancer Research Center, Seattle, WA 98105, USA; 3 Department of Genetics, University of North Carolina, Chapel Hill, NC 27599, USA; 4 Jackson Heart Study, Jackson, MS 39216, USA; 5 Department of Pathology and Laboratory Medicine, University of Vermont Larner College of Medicine, Burlington, VT 05405, USA; 6 Division of Biostatistics, Institute for Health and Equity, and Cancer Center, Medical College of Wisconsin, Milwaukee, WI 53226, USA

**Keywords:** Sickle Cell, T Lymphocyte, Flow Cytometry, CD4, CD8

## Abstract

Sickle cell trait (SCT) has been associated with alterations in various immune-related laboratory parameters including lower circulating lymphocyte counts. To further characterize the impact of SCT on the immune system, we performed flow cytometry of monocyte and lymphocyte immune cell subsets from peripheral blood mononuclear cells collected in a large, community-based cohort of SCT-positive (n = 68) and SCT-negative (n = 959) Black adults. SCT was significantly associated with lower proportions of CD8^+^ and CD4^+^ T cell subsets that include senescent-like markers of repeated immune system challenges. These immune alterations could have potential implications for the susceptibility of individuals with SCT to various infectious diseases.

## 1. Introduction

Sickle cell trait (SCT) is characterized by the inheritance of a single copy of the Hb S mutation and affects, among other populations, approximately 10% of Black individuals in the United States [1,2]. While SCT traditionally has been considered a benign carrier state, it has become increasingly apparent that individuals with SCT may experience subclinical low-grade hemolysis and tend to have lower red cell-related laboratory values (e.g., hemoglobin, red cell count, mean corpuscular volume (MCV)) compared to age-and race-matched Hb A/A individuals [3]. In addition, SCT has been associated with an increased risk of various conditions including exertional rhabdomyolysis [4], chronic kidney disease (CKD) [5], diabetes [6], and venous thromboembolic disease [7]. In particular, SCT has been associated with several kidney complications, including impaired urinary concentration, hematuria/papillary necrosis, lower eGFR, albuminuria, and higher risk of chronic kidney disease (CKD) and progression to end-stage kidney disease (ESKD) [8]. More recently, SCT has been associated with alterations in various immune-related parameters including higher absolute counts and proportion of neutrophils, lower lymphocyte counts and proportion [9], and higher circulating levels of inflammatory proteins such as fractalkine/CX3CL1 [10]. These immune alterations could have potential implications for the recently reported associations of SCT with susceptibility to various infectious diseases [11–15]. To further characterize the impact of SCT on the adaptive and innate immune responses, we performed flow cytometry of monocyte and lymphocyte immune cell subsets from peripheral blood mononuclear cells (PBMC) collected in a large, community-based cohort of SCT-positive and SCT-negative Black adults.

## 2. Methods

The Jackson Heart Study (JHS) is a community-based, longitudinal cohort study designed to identify CVD risk factors among self-identified Black or African American adults age ≥ 21 years from the Jackson, Mississippi metropolitan area, as previously described [16]. Flow cytometry was performed using peripheral blood mononuclear cells (PBMCs) isolated from blood collected and cryopreserved from 1028 JHS participants during the baseline examination (2000–2004). All study sites obtained informed consent and institutional review board approval.

### 2.1. Data Collection

Baseline socio-demographic and medical history were collected using standardized questionnaires. To determine sickle cell mutation status, genotyping for *HBB* rs334 was derived from TOPMed whole genome sequencing (WGS), as previously described [17]. Of 1028 JHS participants with flow cytometry data, there were 68 with SCT (genotyped as Hb A/S) and 959 genotyped as Hb A/A. One individual was genotyped as Hb S/S and was excluded from further analysis. African genetic ancestry was estimated from WGS as the top genotype principal component (PC) using PC-AiR [18]. A complete blood cell count (CBC) and leukocyte differential were performed during the baseline exam using an automated electronic cell counter. Serum creatinine was measured at baseline using an enzymatic method that was traceable to an isotope dilution mass spectrometry reference creatinine standard. Glomerular filtration rate was estimated (eGFR) from serum creatinine using the CKD Epidemiology Collaboration (CKD-EPI) equation [19].

### 2.2. Flow Cytometry

Cell phenotyping assay methods, reagents, and flow cytometry gating strategies for lymphocytes and monocytes have been described in detail [18]. In general, cryopreserved cells were thawed, washed, stained for viability and surface labeled for most phenotypes. For regulatory cells, the surface labeled cells were further explored by intracellular staining for transcription factors. For stimulation assays (i.e., Th1, Th2, Th17), surface-labeled cells were stimulated with PMA/Ionomycin to determine intracellular production of cytokines. Data were collected on a Miltenyi MACSQuant 16 flow cytometer using single-color controls to set compensation and isotype controls to set negative gates and analyzed using FCS Express software. Immune cell phenotypes were expressed as proportions of their parent populations. Details on all the phenotypes measured, their parent populations and their gating strategies were as previously described [20].

### 2.3. Statistical Analysis

To assess the association of immune cell (lymphocyte or monocyte) subset proportions with SCT (Hb A/S) versus non-SCT African American controls (Hb A/A), we performed linear regression with rank-based normal transformed cell proportion as the dependent variable and SCT as the independent variable, adjusting for baseline age, sex, and African genetic ancestry. In total, 141 different immune cell phenotypes were evaluated for association with SCT (Supplemental Table S1). Notably, many of the T cell, B cell, and monocyte phenotypes exhibit varying degrees of correlation with one another. Therefore, to adjust for multiple testing and control the family-wise error rate at an alpha = 0.05, we utilized a resampling-based method that implicitly accounts for the dependence structure of the individual test statistics [21]. Within the JHS flow cytometry dataset, there were 164 families with two or more members, encompassing 793 of the 1027 participants. Therefore, we also conducted regression analyses accounting for familial relationships using mixed linear models assuming a correlation structure within families. Since there were no substantive differences between the results of regression models accounting for family structure versus those not accounting for family structure, we present the results of the latter. In additional sensitivity analyses, we added other covariates (hemoglobin, eGFR, total lymphocyte count) to the SCT-immune cell phenotype association regression models to assess whether the associations are robust to additional adjustment for phenotypes known to be associated with SCT status.

## 3. Results

Among the 1027 participants included in the study, 48% were female and the average age was 62 years (range 40 to 88 years). There were 68 with SCT and 959 Black participants without SCT. The baseline characteristics of the participants are shown in Table 1, stratified by SCT status. Compared to Black individuals without SCT (Hb A/A), those with SCT (HbA/S) had lower age- and sex-adjusted estimated glomerular filtration rate (eGFR), MCV, total white blood cell (WBC) count, and total lymphocyte count (all *p* < 0.05).

In age-, sex-, and genetic ancestry-adjusted linear regression models, SCT was significantly associated with lower proportions of CD8^+^ and CD4^+^ T cell subsets that included CD57^+^, CD27^−^, and/or CD28^−^ (all senescent-like markers of repeated immune system challenges) and the CD8^+^Granzyme B^+^ cells (all adjusted *p* < 0.05) (Table 2).

**Figure 1.**
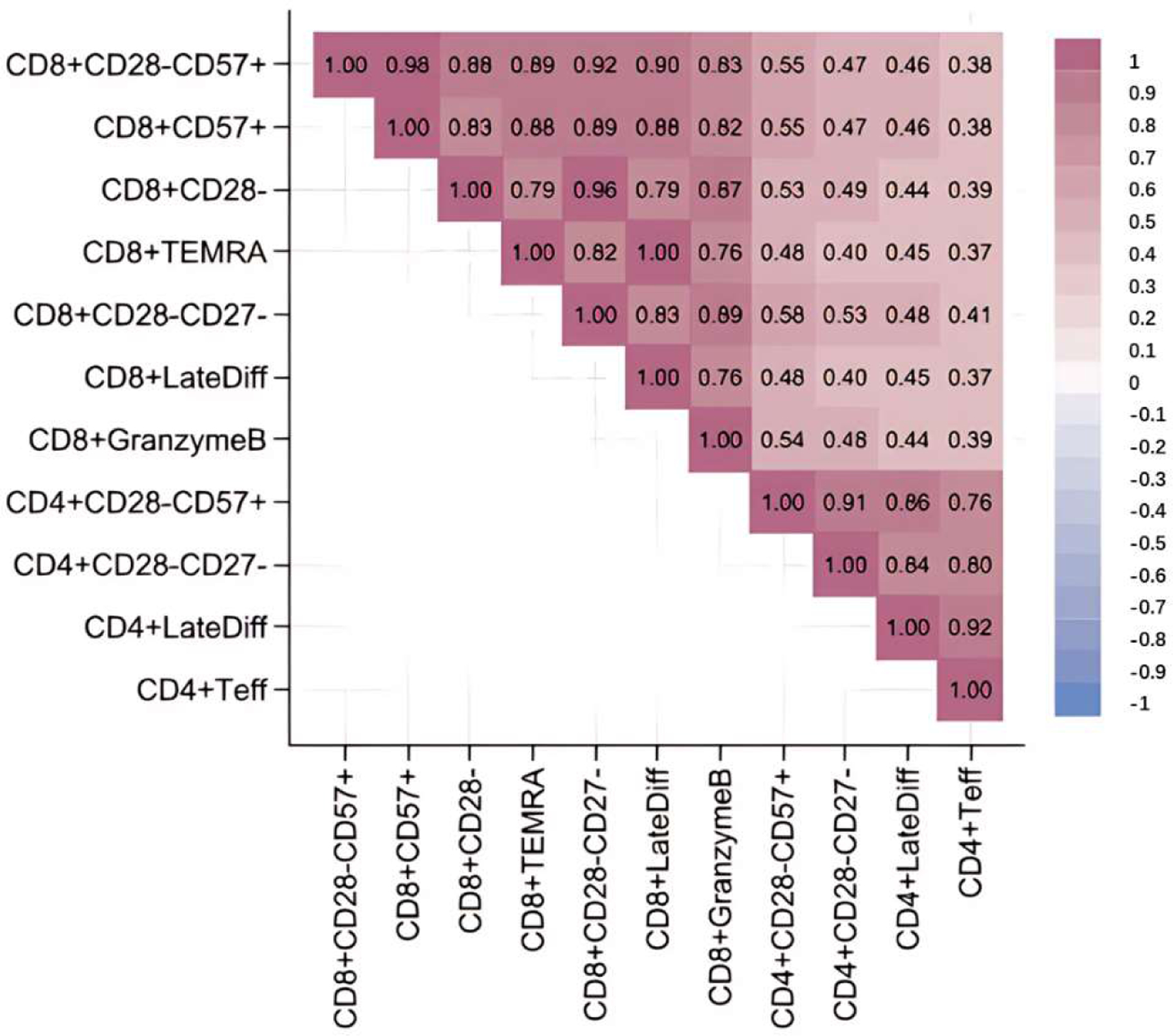
Heatmap of pairwise correlations between SCT-associated lymphocyte subsets.

There was little evidence of association between SCT and proportions of B cells, T regulatory cells, CD4^+^ or CD8^+^ stimulated cells, gamma delta T cells, natural killer cells, or monocyte subsets (Supplemental Table S2). The full set of results and their comparison when accounting for versus not accounting for family structure is shown in Supplemental Table S2. In sensitivity analyses, none of the significant T cell subset-SCT associations were altered by additional adjustment for other phenotypes known to be associated with SCT status (hemoglobin, eGFR, and total lymphocyte count) (Table 2). A heat map of pairwise correlation coefficients between the 12 significant SCT-associated T cell phenotypes shows two main clusters consisting of CD4^+^ versus CD8^+^ subsets (Figure 1).

Spearman pairwise correlations are shown for each of the twelve CD4^+^ or CD8^+^ lymphocyte subsets significantly associated with sickle cell trait in Table 2.

## 4. Discussion

Our current analysis was motivated by the recent finding that SCT is associated with lower total circulating lymphocyte count [9]. The reduced proportions of senescent and differentiated or proliferative CD4^+^ and CD8^+^ T cells with effector functions we observed among individuals with SCT could be due to reduced proliferative capacity, activation, and/or reduced survival. The overall pattern we observed in this community-based African American sample appears to suggest a state of immune inactivation. These immune alterations may have implications for the clinical consequences and pathobiology of SCT, as discussed further below.

Among individuals with SCT, chronic low-grade hemolysis may lead to saturation of heme scavenging mechanisms, resulting in elevated serum levels of free heme. Extracellular heme can trigger a type 1 interferon inflammatory response and activate the NLRP3 inflammasome, which can have downstream consequences for both innate and adaptive immune systems [22,23]; these include recruitment of innate immune cells to sites of inflammation, polarization of macrophages to the pro-inflammatory state, production of interferon-γ, and modulation of T cell responses. Through modulation of heme oxygenase-1 expression, chronic hemolysis may additionally inhibit effector T-cell responses [22].

The pathophysiology and molecular mechanisms underlying the association of SCT with various clinical sequelae remain incompletely understood. Local conditions within the renal medulla and vasa recta (low oxygen tension, reduced blood flow, acidosis, and hyperosmolarity) can promote HbS polymerization and sickling, which likely contribute to SCT susceptibility to kidney disorders. Dysregulation of the adaptive immune system is a common feature of kidney diseases in general, including progression to ESKD [24]. Therefore, the circulating T cell alterations observed in current study may in part reflect local tissue damage in the kidneys and additionally play a role in SCT-related nephropathy. Moreover, the SCT-associated adaptive immune alterations (e.g., reduced proportions of T effector cells) may contribute to the susceptibility of SCT individuals to infectious diseases. SCT has been associated with increased risk of COVID-19 [11], post-operative infection [14], and pneumonia [15]. Future studies that assess the impact of modifiers of Hb S polymerization (such as Hb F levels) [25] on immune function and susceptibility to infection among individuals with SCT may be warranted.

The immune system also has been recognized to play an important role in pathophysiology of sickle cell disease (SCD) and its complications [26]. The release of free heme during intravascular hemolysis leads to release of proinflammatory cytokines, activation of innate immunity, and chronic inflammation, all of which contribute to vaso-occlusive crises [27]. Compared to the innate immune system, changes in adaptive immune response in SCD have been less well characterized. Alterations in T cell subsets have been reported in relatively small numbers of SCD individuals, either in the steady-state or during vaso-occlusive crises, with conflicting results [28–31]. Interestingly, a pattern of alterations in T cell subsets including reduced CD28^−^ effector T cells similar to that observed in the current SCT study was reported in a group of SCD patients in acute crisis state compared to steady-state SCD patients [29]. In contrast, another study reported higher CD4^+^CD28^−^ differentiated effector memory cells among steady-state SCD patients compared to controls and an association of higher CD4^+^CD28^−^ cells in SCD patients with a history of vaso-occlusive crises [31]. Additional investigation of the adaptive immune response and the relationship of lymphocyte phenotypes to clinical outcomes among individuals with either SCD or SCT may identify therapeutic targets for vaso-occlusive crises and susceptibility to infection.

## Supplementary Material

Supplementary Materials

## Figures and Tables

**Table 1.** Baseline characteristics of study participants.

Characteristic	Sickle Cell Trait (SCT)	Non-SCT
N	68	959
Male, n (%)	29 (41)	346 (35)
Age (years)	51.3 ± 14.4	50.1 ± 13.6
Proportion African genetic ancestry, (%)	86	87
Body mass index (kg/m^2^)	30.9 ± 8.0	32.3 ± 7.8
Current smoking, (%)	11.4	13.2
Hypertension, (%)	57.1	54.9
Diabetes, n (%)	15.7	18.1
eGFR, (mL/min/1.73mm^2^) *	83.5 ± 19.9	88.9 ± 19.7
C-reactive protein, (mg/dL)	0.47 ± 0.59	0.57 ± 0.93
Hemoglobin, (g/dL)	13.0 ± 1.4	13.0 ± 1.5
MCV, (fl) *	83.9 ± 6.6	87.0 ± 6.5
WBC, (cells ×10^−9^) *	5.2 ± 1.7	5.8 ± 2.0
Neutrophils, (%)	55.2	53.9
Monocytes, (%)	7.1	7.1
Lymphocytes (%)	34.5	36
Absolute neutrophil count (cells ×10^−9^)	2.90 ± 1.17	3.20 ± 1.57
Absolute monocyte count (cells ×10^−9^)	0.37 ± 0.16	0.39 ± 0.14
Absolute lymphocyte count (cells ×10^−9^) *	1.78 ± 0.66	2.01 ± 0.67

Data are presented as mean ± SD, number (percentage).

* *p* < 0.05 for age and sex-adjusted comparison.

**Table 2.** Significant associations of sickle cell trait with immune cell phenotypes.

Markers	Phenotype	N	Model 1 *				Model 2 **			
			Beta	SE	*p*-Value	Adjusted *p*-Value	Beta	SE	*p*-Value	Adjusted *p*-Value
CD8^+^CD28^−^CD57^+^	CD8 senescent cell	1018	−0.612	0.122	5.68 × 10^−7^	2.00 × 10^−4^	−0.642	0.125	3.09 × 10^−7^	3.00 × 10^−4^
CD8^+^CD57^+^	CD8 senescent cell	1018	−0.591	0.121	1.29 × 10^−6^	6.00 × 10^−4^	−0.621	0.124	6.77 × 10^−7^	7.00 × 10^−4^
CD8^+^CD45RA^+^CD28^−^CD57^+^	CD8 TEMRA	1018	−0.575	0.122	2.91 × 10^−6^	0.0037	−0.6	0.125	2.04 × 10^−6^	0.0037
CD8^+^CD28^−^	CD8 senescent cell	1018	−0.563	0.122	4.14 × 10^−6^	0.0015	−0.604	0.125	1.49 × 10^−6^	4.00 × 10^−4^
CD8^+^CD28^−^CD27^−^	CD8 senescent cell	1018	−0.54	0.12	7.13 × 10^−6^	0.0045	−0.58	0.122	2.38 × 10^−6^	0.0016
CD8^+^CD45RA^+^CD27^−^CD28^−^CD57^+^	CD8 late differentiated	1018	−0.535	0.122	1.33 × 10^−5^	0.0099	−0.557	0.125	9.97 × 10^−6^	0.0115
CD8^+^Granzyme B^+^	Granzyme B-producing CD8	1026	−0.518	0.119	1.47 × 10^−5^	0.0061	−0.574	0.122	2.81 × 10^−6^	0.0016
CD8^+^CD45RO^−^CCR7^−^CD27^−^	CD8 effector cell	1018	−0.513	0.12	1.97 × 10^−5^	0.025	−0.544	0.122	8.61 × 10^−6^	0.0171
CD4^+^CD28^−^CD57^+^	CD4 senescent cell	1018	−0.513	0.123	3.18 × 10^−5^	0.01	−0.537	0.126	2.12 × 10^−5^	0.007
CD4^+^CD28^−^CD27^−^	CD4 senescent cell	1018	−0.495	0.123	5.91 × 10^−5^	0.012	−0.523	0.124	2.81 × 10^−5^	0.0094
CD4^+^CD45RO^−^CCR7^−^CD27^−^	CD4 effector cell	1018	−0.47	0.126	0.000194	0.021	−0.504	0.127	8.09 × 10^−5^	0.0152
CD4^+^CD45RA^+^CD27^−^CD28^−^CD57^+^	CD4 late differentiated	1018	−0.454	0.124	0.000253	0.047	−0.491	0.126	0.000102	0.0287

SE, standard error; TEMRA = CD45RA-re-expressing effector memory cell.

* Model 1 adjusted for baseline age, sex, and African genetic ancestry.

** Model 2 adjusted for baseline age, sex, African genetic ancestry, hemoglobin, estimated glomerular filtration rate (eGFR), and total lymphocyte count.
