# Supplementary material for: Alterations of T Cell Subsets Associated with Sickle Cell Trait
Source: Blood Genom Discov. Author manuscript; Available in PMC 2025 Jan 1. (PMC11665108; doi:10.70322/bgd.2025.10001)
Supplement: Supplementary Materials [file NIHMS2037140-supplement-Supplementary_Materials.pdf]

Supplementary Table S1. Immune phenotype characterization.		
**Phenotype**	**Comment or Defining Markers**	**Parent Population**
**T cells**	CD3+	Live Lymphs
**T helper cells**	CD4+	CD3+
**Naïve-type CD4 cell**	CD4+CD45RA+	CD4+
**Memory CD4 cell**	CD4+CD45RO+	CD4+
**CD4+CD28-**	senescent cell	CD4+
**CD4+CD57+**	senescent cell	CD4+
**CD4+CD95+**		CD4+
**CD4+CD127+**	long-lived memory CD4 cell	CD4+
**CD4+CD137+**		CD4+
**CD4+CD27-**		CD4+
**CD4+PD1+**	Immune checkpoint marker	CD4+
**CD4+CD28-CD57+**	senescent cell	CD4+
**CD4+CD28-CD27-**	senescent cell	CD4+
**CD4 NAÏVE**	CD4+CD45RA+CD27+CD28+CD57-	CD4+
**CD4 ED (early differentiated)**	CD4+CD45RA-CD27+CD28+CD57-	CD4+
**CD4 ID (intermediate differentiated)**	CD4+CD45RA-CD27+CD28-CD57+	CD4+
**CD4 LD (late differentiated)**	CD4+CD45RA+CD27-CD28-CD57+	CD4+
**CD4+ TEMRA**	CD4+CD45RA+CD28-CD57+	CD4+
**CD4 Tn (True naïve)**	CD4+CD45RO-CCR7+CD27+	CD4+
**CD4 Tint (intermediate memory)**	CD4+CD45RO-CCR7-CD27+	CD4+
**CD4 Teff (effector)**	CD4+CD45RO-CCR7-CD27-	CD4+
**CD4 Tcm (central memory)**	CD4+CD45RO+CCR7+CD27+	CD4+
**CD4 Ttm (transitional memory)**	CD4+CD45RO+CCR7-CD27+	CD4+
**CD4 Tem (effector memory)**	CD4+CD45RO+CCR7+CD27-	CD4+
**Cytotoxic T cells**	CD8+	CD3+
**Naïve-type CD8 cells**	CD8+CD45RA+	CD8+
**Memory CD8 cell**	CD8+CD45RO+	CD8+
**CD8+CD28-**		CD8+
**CD8+CD57+**		CD8+
**CD8+CD95+**		CD8+
**CD8+CD127+**		CD8+
**CD8+CD137+**		CD8+
**CD8+CD27-**		CD8+
**CD8+PD1+**	Cancer marker	CD8+
**CD8+CD28-CD57+**	senescent cell	CD8+
**CD8+CD28-CD27-**	senescent cell	CD8+
**CD8 NAÏVE**	CD8+CD45RA+CD27+CD28+CD57-	CD8+

**CD8 ED**	CD8+CD45RA-CD27+CD28+CD57-	CD8+
**CD8 ID**	CD8+CD45RA-CD27+CD28-CDC57+	CD8+
**CD8 LD**	CD8+CD45RA+CD27-CD28-CD57+	CD8+
**CD8+ TEMRA**	CD8+CD45RA+CD28-CD57+	CD8+
**CD8 Tn**	CD8+CD45RO-CCR7+CD27+	CD8+
**CD8 Tint**	CD8+CD45RO-CCR7-CD27+	CD8+
**CD8 Teff**	CD8+CD45RO-CCR7-CD27-	CD8+
**CD8 Tcm**	CD8+CD45RO+CCR7+CD27+	CD8+
**CD8 Ttm**	CD8+CD45RO+CCR7-CD27+	CD8+
**CD8 Tem**	CD8+CD45RO+CCR7+CD27-	CD8+
**T cells**	CD3+	Live Lymphs
**B cells**	CD19+	Live Lymphs
**gd T cells**	gamma delta T cells	CD3+
**NK cells**	CD3-CD56+CD16+	Live Lymphs
**CD38+ T cells**	broad activation marker	CD3+
**HLA-DR+ T cells**	broad activation marker	CD3+
**CD38+HLADR+ T cells**	Activated T cells	CD3+
**IgD B cells**		B cells
**IgM B cells**		B cells
**IgDIgM Bcells**		B cells
**CD43+ B cells**		B cells
**CD27+ B cells**		B cells
**CD43+CD27+ B1**	B1 cells	B cells
**CD43-CD27+ Bmem**	memory B cells	B cells
**IgD B1cell**		B1 cells
**IgM B1cell**		B1 cells
**IgD IgM B1cell**		B1 cells
**IgD Bmem**		B mem Cells
**IgM Bmem**		B mem Cells
**IgD IgM Bmem**		B mem Cells
**CD3_ICS**	T cells	Live Lymphs
**CD4_ICS**	T helper cells	CD3+
**CD8_ICS**	Cytotoxic T cells	CD3+
**TH1**	CD4+IFNg+	CD4+
**TH2**	CD4+IL4+	CD4+
**TH17**	CD4+IL17+	CD4+
**CD4+TNFa+**		CD4+
**GRANZYME B + CD4**		CD4+
**PERFORIN+ CD4**		CD4+
**TNFa+IFNg+ CD4**		CD4+

**IL17+IFNg+ CD4**		CD4+
**IL17+IFNg+ TH1**		Th1
**Tc1**	CD8+IFNg+	CD8+
**Tc2**	CD8+IL4+	CD8+
**Tc17**	CD8+IL17+	CD8+
**CD8+TNFa+**		CD8+
**Granzyme B+ CD8**		CD8+
**PERFORIN+ CD8**		CD8+
**TNFa+IFNg+ CD8**		CD8+
**IL17+IFNg+ CD8**		CD8+
**IL17+IFNg+ Tc1**		Tc1
**CD3_regs**	T cells	Live Lymphs
**CD4_regs**	T helper cells	CD3+
**CD8_regs**	Cytotoxic T cells	CD3+
**CD4+CD25+**	Broad regulatory cell	CD4+
**CD4+CD25+CD127-**	CD4+ REGULATORY T CELL	CD4+
**CD4+CD25+CD127-FoxP3+**		CD4+
**CD4+CD25+CD127-Tbet+**		CD4+
**CD4+CD25+CD127-FoxP3+Tbet+**		CD4+
**CD4+CD25+CD127-Rorgt+**		CD4+
**CD4+CD25+CD127-FoxP3+Rorgt+**		CD4+
**CD4+FoxP3+**		CD4+
**CD4+CD25+FoxP3+**	FoxP3 CD4 TREGS	CD4+
**CD4+Tbet+**		CD4+
**CD4+Rorgt+**		CD4+
**CD4+Tbet+Rorgt+**		CD4+
**CD8+CD25+**		CD4+
**CD8+CD25+CD127-**		CD4+
**CD8+CD25+CD127-FoxP3+**		CD4+
**CD8+CD25+CD127-Tbet+**		CD4+
**CD8+CD25+CD127-FoxP3+Tbet+**		CD4+
**CD8+CD25+CD127-Rorgt+**		CD4+
**CD8+CD25+CD127-FoxP3+Rorgt+**		CD4+
**CD8+FoxP3+**		CD4+
**CD8+CD25+FoxP3+**		CD4+
**CD8+Tbet+**		CD4+
**CD8+rORGT+**		CD4+
**CD8+Tbet+Rorgt+**		CD4+

**CD14 total**		Gated monos
**CLASSICAL MONOCYTES (CM)**	CD14++CD16-	CD14
**INTERMEDIATE MONOCYTES (IM)**	CD14+CD16+	CD14
**NONCLASSICAL MONOCYTES (NCM)**	CD14dimCD16+	CD14
**HLADR+ CM**		CM
**TREM2+ CM**		CM
**CD87+ CM**		CM
**CCR2+ CM**		CM
**Slan+ CM**		CM
**CD33+ CM**		CM
**CD163+ CM**		CM
**HLADR+ IM**		IM
**TREM2+ IM**		IM
**CD87+ IM**		IM
**CCR2+ IM**		IM
**Slan+ IM**		IM
**CD33+ IM**		IM
**CD163+ IM**		IM
**HLADR+ NCM**		ncm
**TREM2+ NCM**		NCM
**CD87+ NCM**		NCM
**CCR2+ NCM**		NCM
**Slan+ NCM**		NCM
**CD33+ NCM**		NCM
**CD163+ NCM**		NCM

Supplemental Table S2. Association of sickle cell trait with immune cell phenotypes, adjusting for age, sex, and genetic ancestry

Phenotype	Not accounting for relatedness			Accounting for relatedness		
	Beta	SE	p-value	Beta	SE	p-value
cd8cd28cd57	-0.612	0.122	5.68E-07	-0.586	0.123	1.88E-06
cd8cd57	-0.591	0.121	1.29E-06	-0.556	0.123	5.78E-06
cd8temra	-0.575	0.122	2.91E-06	-0.546	0.124	1.00E-05
cd8cd28	-0.563	0.122	4.14E-06	-0.547	0.123	8.31E-06
cd8cd28cd27	-0.540	0.120	7.13E-06	-0.517	0.121	1.93E-05
cd8ld	-0.535	0.122	1.33E-05	-0.512	0.124	3.46E-05

granzymebcd8	-0.518	0.119	1.47E-05	-0.481	0.121	6.63E-05
cd8teff	-0.513	0.120	1.97E-05	-0.484	0.121	6.46E-05
cd4cd28cd57	-0.513	0.123	3.18E-05	-0.475	0.124	1.31E-04
cd4cd28cd27	-0.495	0.123	5.91E-05	-0.471	0.124	1.38E-04
cd4teff	-0.470	0.126	1.94E-04	-0.438	0.127	5.45E-04
cdld	-0.454	0.124	2.53E-04	-0.422	0.125	7.13E-04
cd8cd25cd127tbet	-0.437	0.122	3.60E-04	-0.394	0.124	1.47E-03
cdid	-0.415	0.119	5.32E-04	-0.412	0.121	6.43E-04
cd4cd57	-0.408	0.123	9.72E-04	-0.358	0.125	4.06E-03
cd4temra	-0.411	0.125	1.01E-03	-0.368	0.126	3.51E-03
perforincd8	-0.387	0.121	1.43E-03	-0.336	0.121	5.35E-03
trem2cm	0.444	0.141	1.67E-03	0.396	0.143	5.66E-03
cd4t	-0.393	0.125	1.69E-03	-0.391	0.126	1.89E-03
tc1	-0.359	0.117	2.10E-03	-0.338	0.117	4.01E-03
il17ifngth1	0.374	0.123	2.43E-03	0.353	0.124	4.54E-03
cd4cd28	-0.377	0.125	2.60E-03	-0.348	0.126	5.73E-03
cd8cd127	0.371	0.124	2.80E-03	0.344	0.125	6.12E-03
trem2im	0.410	0.140	3.46E-03	0.392	0.142	5.73E-03
cd4tem	-0.356	0.122	3.70E-03	-0.374	0.124	2.44E-03
th17	0.364	0.125	3.73E-03	0.351	0.127	5.70E-03
cd8cd27	-0.330	0.115	4.15E-03	-0.307	0.116	8.46E-03
cd8tn	0.310	0.112	5.68E-03	0.289	0.113	1.08E-02
tnfaifngcd8	-0.322	0.116	5.70E-03	-0.299	0.117	1.09E-02
trem2ncm	0.374	0.139	7.14E-03	0.388	0.142	6.15E-03
cd8id	-0.308	0.114	7.21E-03	-0.322	0.115	5.35E-03
cd4cd27	-0.326	0.124	8.56E-03	-0.310	0.125	1.30E-02
th1	-0.326	0.124	8.69E-03	-0.300	0.125	1.67E-02
cd43cd27bmem	-0.329	0.127	9.54E-03	-0.276	0.125	2.74E-02
granzymebcd4	-0.315	0.122	9.72E-03	-0.299	0.123	1.53E-02
cd4cd127	0.313	0.126	1.31E-02	0.297	0.127	1.94E-02
cd8naive	0.265	0.113	1.94E-02	0.255	0.115	2.64E-02
cd163im	0.314	0.141	2.64E-02	0.286	0.144	4.73E-02
il17ifngtc1	0.325	0.146	2.66E-02	0.311	0.147	3.44E-02
cd27bcells	-0.267	0.127	3.56E-02	-0.212	0.126	9.13E-02
cd8cd25cd127foxp3tbet	-0.217	0.105	3.86E-02	-0.192	0.107	7.23E-02
icscd3	-0.255	0.125	4.07E-02	-0.238	0.126	5.96E-02
cd8tbet	-0.255	0.125	4.19E-02	-0.226	0.127	7.49E-02
il17ifngcd4	0.251	0.124	4.22E-02	0.239	0.125	5.62E-02
cd87im	-0.124	0.063	4.80E-02	-0.128	0.063	4.32E-02
cd8tcm	0.228	0.124	6.68E-02	0.203	0.126	1.07E-01
th2	0.228	0.125	6.80E-02	0.247	0.126	5.00E-02

bcells	-0.226	0.125	7.09E-02	-0.244	0.126	5.19E-02
tc17	0.221	0.123	7.36E-02	0.225	0.124	7.02E-02
im	-0.251	0.141	7.60E-02	-0.221	0.142	1.21E-01
cd4n	0.213	0.121	7.84E-02	0.222	0.122	6.95E-02
cd4pd1	-0.220	0.127	8.20E-02	-0.253	0.128	4.76E-02
cd33	-0.244	0.141	8.38E-02	-0.190	0.141	1.77E-01
igdbcells	0.217	0.126	8.58E-02	0.162	0.128	2.06E-01
cd163ncm	0.239	0.140	8.87E-02	0.241	0.141	8.72E-02
cd3	-0.206	0.125	9.89E-02	-0.177	0.126	1.62E-01
cd4naive	0.197	0.121	1.04E-01	0.206	0.123	9.34E-02
slanim	0.225	0.139	1.07E-01	0.220	0.142	1.20E-01
cd8cd25cd127foxp3rorgt	-0.174	0.109	1.11E-01	-0.203	0.111	6.75E-02
cd8cd25cd127foxp3	-0.176	0.111	1.13E-01	-0.194	0.112	8.43E-02
slancm	0.222	0.141	1.16E-01	0.206	0.144	1.51E-01
tnfaifngcd4	-0.195	0.124	1.16E-01	-0.165	0.126	1.89E-01
cd43bcells	0.196	0.125	1.18E-01	0.178	0.126	1.59E-01
cd8cd25cd127	-0.194	0.126	1.23E-01	-0.195	0.127	1.26E-01
tregscd8	-0.195	0.127	1.25E-01	-0.211	0.129	1.02E-01
cd8cd137	-0.192	0.127	1.30E-01	-0.206	0.124	9.73E-02
ccr2ncm	0.204	0.138	1.40E-01	0.115	0.141	4.15E-01
tcells	-0.180	0.125	1.50E-01	-0.126	0.126	3.18E-01
cd8tnfa	-0.162	0.112	1.50E-01	-0.152	0.113	1.79E-01
icscd8	-0.179	0.125	1.53E-01	-0.194	0.127	1.26E-01
cd8ed	0.179	0.126	1.54E-01	0.153	0.127	2.27E-01
ccr2cm	-0.193	0.140	1.67E-01	-0.208	0.143	1.44E-01
cd33cm	-0.191	0.142	1.78E-01	-0.180	0.143	2.09E-01
cd31	-0.167	0.124	1.80E-01	-0.141	0.126	2.63E-01
cd8tem	-0.163	0.122	1.81E-01	-0.170	0.123	1.67E-01
cd8tbetrorgt	-0.164	0.124	1.87E-01	-0.119	0.126	3.43E-01
gdtcells	-0.163	0.125	1.90E-01	-0.137	0.126	2.76E-01
cd87ncm	0.167	0.128	1.93E-01	0.090	0.130	4.89E-01
cd33ncm	-0.176	0.141	2.10E-01	-0.153	0.142	2.83E-01
tregscd3	-0.159	0.127	2.13E-01	-0.116	0.129	3.69E-01
perforincd4	-0.153	0.124	2.18E-01	-0.117	0.125	3.49E-01
icscd4	0.149	0.124	2.31E-01	0.153	0.125	2.23E-01
cm	0.167	0.141	2.38E-01	0.120	0.144	4.02E-01
cd4cd25cd127	0.146	0.127	2.51E-01	0.091	0.129	4.78E-01
hladrncm	0.144	0.125	2.51E-01	0.084	0.128	5.13E-01
cd8cd25cd127rorgt	-0.140	0.125	2.64E-01	-0.145	0.127	2.54E-01
cd4tbet	-0.138	0.126	2.74E-01	-0.115	0.128	3.69E-01
cd4cd137	-0.139	0.127	2.75E-01	-0.151	0.127	2.37E-01

cd4ra	0.132	0.121	2.78E-01	0.157	0.122	1.97E-01
tc2	0.129	0.125	3.05E-01	0.150	0.126	2.37E-01
cd163cm	0.141	0.138	3.07E-01	0.107	0.139	4.44E-01
cd4cd25cd127rorgt	0.126	0.126	3.18E-01	0.084	0.127	5.12E-01
igdbmem	0.122	0.127	3.34E-01	0.069	0.128	5.90E-01
cd8	-0.121	0.126	3.36E-01	-0.119	0.127	3.48E-01
nkcells	0.116	0.121	3.41E-01	0.117	0.123	3.42E-01
cd4ro	-0.116	0.122	3.42E-01	-0.140	0.123	2.54E-01
cd4tnfa	-0.112	0.122	3.58E-01	-0.123	0.124	3.20E-01
cd87cm	-0.118	0.139	3.96E-01	-0.110	0.141	4.34E-01
cd4cd25foxp3	-0.105	0.125	4.01E-01	-0.116	0.126	3.57E-01
cd8t	-0.104	0.126	4.09E-01	-0.084	0.127	5.09E-01
il17ifngcd8	0.118	0.146	4.18E-01	0.109	0.147	4.56E-01
cd4ttm	-0.100	0.126	4.26E-01	-0.152	0.127	2.34E-01
cd8cd95	0.090	0.119	4.50E-01	0.086	0.121	4.76E-01
cd4tbetrorgt	-0.083	0.126	5.06E-01	-0.056	0.127	6.62E-01
igdb1	0.085	0.128	5.07E-01	0.074	0.129	5.66E-01
slanncm	0.088	0.139	5.29E-01	0.099	0.140	4.80E-01
cd14total	0.086	0.141	5.41E-01	0.060	0.143	6.75E-01
igdigmbcells	0.075	0.126	5.48E-01	0.029	0.126	8.20E-01
cd38tcells	-0.071	0.125	5.69E-01	0.015	0.126	9.08E-01
cd4rorgt	0.071	0.126	5.74E-01	0.062	0.129	6.32E-01
cd8foxp3	-0.066	0.121	5.83E-01	-0.064	0.123	6.03E-01
hladrcm	0.074	0.140	5.98E-01	0.105	0.143	4.65E-01
cd8cd25foxp3	0.063	0.125	6.12E-01	0.023	0.126	8.57E-01
cd4cd25	0.064	0.128	6.18E-01	0.038	0.130	7.68E-01
cd4cd25cd127foxp3tbet	-0.052	0.111	6.40E-01	-0.057	0.113	6.13E-01
cd4cd25cd127tbet	-0.059	0.126	6.41E-01	-0.059	0.128	6.44E-01
cd4	0.058	0.125	6.43E-01	0.057	0.126	6.50E-01
cd43cd27b1	0.053	0.125	6.71E-01	0.061	0.126	6.27E-01
cd8pd1	-0.044	0.127	7.32E-01	-0.086	0.128	4.98E-01
igmbcells	0.041	0.126	7.48E-01	-0.007	0.127	9.57E-01
ccr2im	-0.037	0.128	7.74E-01	-0.078	0.130	5.52E-01
cd8rorgt	0.035	0.126	7.80E-01	0.018	0.129	8.88E-01
cd8ro	-0.034	0.122	7.82E-01	-0.055	0.123	6.57E-01
igmb1	0.034	0.128	7.92E-01	-0.001	0.130	9.93E-01
tregscd4	0.033	0.127	7.95E-01	0.026	0.129	8.42E-01
igdigmbmem	0.031	0.126	8.08E-01	-0.022	0.127	8.62E-01
cd8cd25	0.029	0.127	8.21E-01	0.007	0.129	9.59E-01
cd8ra	0.027	0.122	8.25E-01	0.039	0.123	7.49E-01
igdigmb1	0.026	0.126	8.35E-01	0.003	0.127	9.80E-01

cd38hladrtcells	0.025	0.121	8.36E-01	0.067	0.119	5.73E-01
igmbmem	0.019	0.127	8.78E-01	-0.028	0.127	8.27E-01
cd4cd95	0.017	0.123	8.89E-01	-0.005	0.125	9.65E-01
cd4cd25cd127foxp3	0.017	0.122	8.92E-01	0.001	0.123	9.94E-01
hladrtcells	0.016	0.120	8.96E-01	0.002	0.118	9.86E-01
hladrim	0.014	0.120	9.04E-01	0.019	0.122	8.74E-01
cd4foxp3	-0.014	0.127	9.14E-01	-0.033	0.129	8.01E-01
cded	-0.008	0.124	9.51E-01	-0.040	0.126	7.51E-01
cd8ttm	0.007	0.126	9.53E-01	-0.012	0.127	9.23E-01
cd4tcm	-0.007	0.123	9.57E-01	-0.018	0.125	8.84E-01
ncm	0.007	0.141	9.60E-01	0.011	0.142	9.38E-01
cd4cd25cd127foxp3rorgt	-0.003	0.120	9.81E-01	-0.049	0.122	6.88E-01
